# Valorization of Beetroot Pomace as a Flour Fortifier, Functional Ingredient and Dietary Supplement

**DOI:** 10.3390/foods15071142

**Published:** 2026-03-26

**Authors:** Stanislava Gorjanović, Ferenc T. Pastor, Darko Micić, Margarita Dodevska, Slavica Ristić, Saša Petričević, Filip Dujmić, Snežana Zlatanović

**Affiliations:** 1 Institute of General and Physical Chemistry, University of Belgrade, Studentski trg 12/V, 11158 Belgrade, Serbia; micic83@gmail.com; 2 Faculty of Chemistry, University of Belgrade, Studentski trg 12-16, 11158 Belgrade, Serbia; fpastor@chem.bg.ac.rs; 3 Institute of Public Health of Serbia “Dr Milan Jovanovic Batut”, Center for Hygiene and Human Ecology, Dr Subotica Starijeg 5, 11000 Belgrade, Serbia; 4 Institute for Medical and Clinical Biochemistry, Faculty of Medicine, University of Belgrade, Pasterova 2, 11000 Belgrade, Serbia; 5 Faculty of Food Technology and Biotechnology, University of Zagreb, Pierottijeva ul. 6, 10000 Zagreb, Croatia

**Keywords:** beetroot, pomace, anti-grain flour, obesity, glycemic index, waste management

## Abstract

The aim of this study was to evaluate the potential of minimally processed beetroot pomace (BP), obtained from an industrial juice producer selected as a case study, converted into a stable beetroot pomace flour (BPF) at an industrial scale level, for flour fortification, functional confectionery development, and dietary supplementation. It was characterized by a high dietary fiber content (~27 g/100 g) and a very low carbohydrate-to-fiber ratio (1.9). High level of total phenolics and flavonoids (14.1 ± 0.1 mg GAE/g and 1.43 ± 0.1 mg QE/g), betacyanins and betaxanthins (898 ± 54 and 960 ± 65 µg/g), as well as pronounced antioxidant (FRAP 31.5 ± 1.1 and DPPH 25.8 ± 2.9 µmol TE/g), anti-hyperglycemic and anti-inflammatory activity (27.3 ± 1.3% and 41.0 ± 3.4%) remained upon in vitro digestion. Replacing 14–28% of cereal and pseudo-cereal flour with BPF reduced the carbohydrate-to-fiber ratio to the recommended 10:1, while incorporation of 20% BPF into cookies reduced this ratio by 2.5-fold and the glycemic index from ~56 to ~30. Furthermore, long-term supplementation of standard and high-fat/high-sucrose diet with BPF (0.5% *w*/*w*) reduced feed efficiency by 1.7 and 2.6-fold respectively, and improved glucose tolerance in C57BL/6J mice. Findings show the effectiveness of the by-product in bridging the fiber intake gap and body weight regulation.

## 1. Introduction

The fruit and vegetable processing industry generates huge amounts of pomace, a valuable by-products rich in bioactive molecules missing in modern diet, commonly treated as waste [1]. The enormous potential of pomace remains underutilized worldwide. In Serbia and the surrounding region, even minimally processed pomace from organically grown fruits and vegetables is composted or dried and used as a fuel. Such an approach represents wasting of valuable resources but allows producers to avoid associated costs imposed by regulatory authorities, as well as conflicts with local communities that occurred due to the disposal at landfills or illegal dumping sites. Implementing the various possible solutions to utilize by-products reduces environmental impact and opens up economic opportunities through their valorization [2].

Since beetroot (*Beta vulgaris* L.), is popular as a functional food with huge health benefits [3], the amount of waste generated by the beetroot processing industry is constantly increasing (leaves, stems, peels, and pomace). Both beetroot and its processing by-products represent valuable sources of dietary fiber, betalains, and phenolic and flavonoid compounds with antioxidant, anti-inflammatory, and antiglycemic activity, as well as potential to be used in the nutraceuticals [3,4]. The antidiabetic potential of beetroot phytochemicals observed in animal models was linked with the modulation of enzymes involved in carbohydrate metabolism (α-amylase and α-glucosidase), insulin secretion, and oxidative stress [5]. In human clinical studies, long-term daily consumption of beetroot products reduced inflammatory markers involved in diabetes 2 pathogenesis, highlighting systemic metabolic effects beyond glucose lowering [6]. A broader systematic review of beetroot products concluded that consumption modulates blood glucose levels in humans across diverse health conditions, but outcomes vary in relation to product type, dosage or study design [7]. These findings support the concept that beetroot pomace is not only a nutrient-dense ingredient but also a promising functional component for metabolic health promotion, including obesity and type 2 diabetes prevention, reinforcing its value for upcycling into functional food formulations. As a consequence, the interest in a sustainable approach for the utilization of minimally processed BP through conversion to value-added products that can improve the economic efficiency of the beetroot processing industry and reduce the pollution caused by landfill disposal also increases.

At the same time, there is growing recognition that incidences of non-communicable diseases (NCD), including obesity and type 2 diabetes, may be mitigated by incorporating dietary fiber and bioactive compounds into the diet. The American Heart Association recommends a carbohydrate-to-fiber ratio of 10:1 as criteria to identify healthy grain foods [8]. The presence of at least 1 g of fiber per 10 g of carbohydrates (ratio carb:fiber ≤ 10:1) is found to be inversely proportional to cardiometabolic risk, obesity, insulin resistance, and dyslipidemia [9]. The content of easily available sugars is significantly lower in pomace than in whole fruits or vegetables resulting in an even lower carbohydrate-to-fiber ratio which is a key element of appropriate body weight (BW) management. On the other side, many commonly consumed cereal-based and bakery products far exceed the 10:1 ratio, reflecting insufficient fiber relative to carbohydrate content. Thus, incorporating fruit and vegetable pomace can provide a sustainable approach to improve the nutritional profile of such foods. By increasing fiber content without substantially adding digestible carbohydrates, pomace-enriched products could reach or approach the recommended ≤10:1 carb:fiber ratio, thus enhancing postprandial glycemic control and supporting BW management. This strategy might valorize industrial by-products from juice production, reducing food waste while simultaneously creating innovative functional ingredients for cereal-based and confectionery products. The anti-obesity potential of apple pomace flour has been demonstrated [10], highlighting the feasibility of a similar approach for beetroot by-products. Incorporation of apple and beetroot pomace into food matrices based on various gelling agents was shown to lower postprandial glucose responses, glycemic index, and load, due to the synergistic action of fiber, polyphenolics, and betalains [11].

This study evaluates the valorization potential of beetroot pomace flour (BPF) obtained at an industrial scale level by technological process that includes dehydration of minimally processed BP from industrial juice production, followed by grinding to the desired particle size. In contrast to most previous studies using BP prepared at the laboratory scale or pomace extracts, this study focuses on BP obtained and stabilized through industrial-scale drying, representing a higher technology readiness level (TRL) that ensures material relevance as well as readiness for commercial-scale applications. The work was conducted as a case study, using pomace from the well-defined production stream of a trustworthy industrial producer. A comprehensive physicochemical and techno-functional characterization was performed, and anti-obesity related components, including dietary fiber, fructans, polyphenolic compounds, and betalains, were determined, along with antioxidant, anti-hyperglycemic, and anti-inflammatory activity. The study presents a versatile approach to BPF utilization through three main avenues: (i) as a component of composite flour or flour fortifier, (ii) as an ingredient in popular confectionery product, and (iii) as a dietary supplement or natural nutraceutical.

## 2. Materials and Methods

### 2.1. Chemicals

KCl, KH_2_PO_4_, NaHCO_3_, NaCl, MgCl_2_ × 6H_2_O, (NH_4_)_2_CO_3_, Na_2_CO_3_, NaNO_2,_ AlCl_3_ × 6H_2_O, CaCl_2_ × 2H_2_O, FeCl_3_, NaOH, HCl and acetone were of p.a. quality and, together with and diclofenac sodium salt, were obtained from Merck, Darmstadt, Germany. Ethanol Ph.Eur was from Zorka Pharma, Šabac, Serbia. Enzymes α-amylase, pepsin and pancreatin, as well as bile salts used for in vitro digestion were from the list of reagents of Infogest protocol and, same as the (+)-catechin hydrate, (-)-epicathechin, vanillic, protocatechuic (3,4-dihydroxybenzoic), 4-hydroxy benzoic, chlorogenic and gallic acid, gentisic (2,5-dihydroxybenzoic) acid sodium salt hydrate, trolox, DPPH (2,2-diphenyl-1-picrylhydrazyl) and Folin–Ciocalteu’s phenol reagent, were obtained from Sigma-Aldrich (Merck) Darmstadt, Germany. 4-nitrophenyl-α-D-glucopyranoside and TPTZ (2,4,6-Tris(2-pyridyl)-s-triazine) were obtained from Tokyo Chemical Industry Co., Ltd., Japan. Ultrapure water was used for all experiments.

### 2.2. Flour Production, Stability and Safety

Beetroot pomace (BP) was obtained at the industrial scale level (Fruvita, Smederevo, Republic of Serbia). No treatment other than squeezing thoroughly washed beetroots was used. Immediately after squeezing BP was collected aseptically and dehydrated applying a recently patented technological process [12]. Water activity (aw) of dehydrated BP was measured by Labswift aw meter Novasina AG, Lachen, Switzerland) upon dehydration and during a year of storage. The low moisture content of 3.4 wt% and aw of approx. 0.2 enabled grinding of the dehydrated BP to particle size 300 µm. Also, low wt% and aw ensured good stability and storability of the resulting beetroot pomace flour (BPF). Prior to analysis, diet supplementation or cookies production BPF was stored in multilayer paper sacks in standard conditions.

Differential scanning calorimetry (DSC) analysis was performed using a Q1000 DSC instrument (TA Instruments, New Castle, DE, USA) in order to evaluate the glass transition temperature (Tg) of BPF. Samples (5–7 mg) were placed in aluminum pans with pierced lids, with an identical empty pan served as the reference, and heated from −90 to 250 °C at a constant rate of 10 °C/min. Measurements were conducted under a nitrogen atmosphere with a purge flow rate of 60 mL/min to maintain inert conditions and reduce oxidative degradation. The Tg value was determined as the midpoint of the heat capacity change (ΔCp) observed in the DSC curves, as described by Zlatanović et al. [13]. Data collection and processing were performed using Universal Analysis 2000 software (TA Instruments).

The safety of BPF was confirmed by the acute oral toxicity test conducted in accordance with OECD Guideline No. 423 (Acute Oral Toxicity—Limit Test) [14]. Via gastric gavage, BPF was administered to each of five experimental animals at a dose of 2 g/kg body weight. The animals were monitored for signs of toxic reactions over a 14-day observation period.

### 2.3. Determination of Proximate Composition

Protein, fat, total, soluble and insoluble dietary fiber, and ash contents of BPF were determined according to standard procedures of the Association of Official Analytical Chemists (AOAC) [15]. Protein content was determined by the Kjeldahl method (AOAC 978.04) using a nitrogen-to-protein conversion factor of 6.25. Fat was determined by Soxhlet extraction with petroleum ether (AOAC 920.39). Total, soluble, and insoluble dietary fiber was determined by the enzymatic–gravimetric procedure (AOAC 991.43). The content of fructans was determined according to the instruction manual (Megazyme, K-FRUCHK, Bray, Ireland) (AOAC 999.03). Ash content was determined by dry ashing in a muffle furnace at 550 ± 25 °C until constant weight was achieved (AOAC 923.03). In addition, cellulose was determined by the standard method SRPS ISO 6541:1997 [16]. All analyses were performed in triplicate and results were expressed on a dry weight basis.

Available carbohydrate content (ACC) was calculated using Equation (1):

(1) ACC(%)=100−(fat(g)+ash(g)+moisture(g)+protein(g)+DF(g))

Determination of sucrose, D-glucose, and D-fructose was performed according to the instruction manual by the spectrophotometric method using the enzymatic assay kit R-biopharm (R-BIOPHARM AG, Darmstadt, Germany), as described previously [11].

Total energy, calculated by multiplying determined carbohydrate, fat, protein, and fiber content with their average energy values (fat 9 kcal/g, protein 4 kcal/g, carbohydrate 4 kcal/g, and fiber 2 kcal/g), was expressed in kcal per 100 g of samples.

### 2.4. Calculation of BPF Required to Reach 10:1 Carbohydrates:Fiber Ratio in Composite Flour

The mass or percentage of anti-grain flour (BPF) required to be added in various grain flour to reach carb:fiber 10:1 was calculated using the following equation:

(2) $$x=\frac{100\times {\mathrm{TCH}}_{F}-1000\times {\mathrm{DF}}_{F}}{10\times {\mathrm{DF}}_{\mathrm{BPF}}\;{-\mathrm{TCH}}_{\mathrm{BPF}}}$$

(3) $$y=\frac{100\times x}{100+x}$$

x—BPF (g) required to be added into 100 g of grain flour to reach carb:fiber ratio 10:1.
y—BPF (%) required in mixture with grain flour to reach carb:fiber ratio 10:1.
TCH_F_—total carbohydrates content in grain flour (g/100 g).
TCH_BPF_—total carbohydrates content in BPF (g/100 g).
DF_F_—dietary fiber content in grain flour (g/100 g).
DF_BPF_—dietary fiber content in BPF (g/100 g).

### 2.5. In Vitro Digestion of BPF

Digestion of BPF in simulated gastrointestinal fluids was conducted according to the adapted Infogest procedure [17]. For mouth simulation, BPF (2 g) was placed in a 50 mL conical tube and mixed with oral fluid (2.5 mL), 1500 U/mL of α-amylase (0.5 mL), 25 μL of 0.3 M CaCl_2_ and 7.0 mL of water. pH was adjusted to 7.0 and the mixture was incubated at 37 °C for 2 min. For stomach simulation, the addition of gastric solution (5 mL) was followed by the addition of 25,000 U/mL pepsin (0.8 mL) and 5 µL of 0.3 M CaCl_2_. Hydrochloric acid (6 M) was used to adjust the pH to 3, 20 mL of water was added and the mixture was incubated for 2 h at 37 °C under the agitation of 200 rpm. For the intestinal stage, 5 mL of intestinal fluid, 5 mL of 800 U/mL pancreatin, 20% (*w*/*v*) bile salts (1 mL), 40 μL of 0.3 M CaCl_2_ 5 M NaOH to adjust pH to 7.4, and water up to the total volume of 40 mL were added. After incubation for 2 h at 37 °C under agitation of 200 rpm, the tubes were kept in an ice bath for 15 min to eliminate intestinal enzymatic activity and centrifuged at 12,000 rpm. The supernatant (bioaccessible fraction) was collected and subjected to further analysis.

### 2.6. Determination of Total Phenolic and Total Flavonoids Content in Digested BPF

The slightly modified procedure developed by Singleton was applied to determine total phenolics content (TPC), as described by Gorjanović et al. [10]. Appropriately diluted samples (0.50 mL) were mixed with Folin–Ciocâlteu (FC) reagent previously diluted with distilled water in a 1:10 ratio (2.50 mL) and allowed to react for 5 min in the dark. Then 2.0 mL of 7.5% sodium carbonate solution was added; the solution was shaken and left to react for two hours in the dark. The absorbance at 760 nm (A_760_) was measured. Distilled water was used as blank. Three different dilutions of each sample were used, and results were averaged and expressed in mg of gallic acid equivalents (GAE) per g of sample (mg GAE/g).

Total flavonoid content was determined by a colorimetric method as described previously [18]. Briefly, the sample (250 µL) was mixed with 1.75 mL of water and 100 µL of 5% NaNO_2_ solution. After 6 min 200 mL of 10% AlCl_3_ × 6H_2_O solution was added and the mixture was allowed to stand for 5 min when 0.67 mL of 1 M NaOH solution and distilled water to 3.33 mL (0.36 mL) was added. The solution was mixed well, and the absorbance was immediately measured at 510 nm against the blank. The results were expressed as mg of catechin equivalents per g of samples.

### 2.7. Determination of Antiradical Activity and Total Reducing Power in Digested BPF

The antiradical activity of samples against DPPH radical was measured by the modified method of Blois [19]. Three different concentrations of diluted samples (0.20 mL) were mixed with 2.80 mL of a DPPH solution (0.1 mM) in a 2:1 (*v*/*v*) mixture of EtOH and 0.1 M acetate buffer pH 4.3. The mixture was shaken and left for 1 h in the dark before the absorbance measurement at 525 nm. The results are expressed as mM of Trolox equivalents per g of digested sample (mM TE/g).

The FRAP assay was performed according to the procedure previously described in [20] with some modifications introduced. The FRAP reagent solution was made by mixing 0.15 M acetate buffer (pH 3.6), TPTZ (10 mM TPTZ solution in 40 mM HCl), and 20 mM FeCl_3_ in a volume ratio of 10:1:1, respectively. Aliquots of diluted supernatant of digested BPF (0.1 mL) were added to distilled water (0.3 mL) and FRAP reagent (3 mL) to form mixtures. After 40 min incubation in the dark absorbance at 593 nm was measured. The results were expressed as mM Trolox equivalent per g of digested sample (mM TE/g).

### 2.8. Determination of Betacyanins (Bc) and Betaxanthins (Bx) Content in Digested BPF

The digested sample (100 µL) was diluted in 5 mL of water and absorbance was measured at 538 and 480 nm for betacyanins and betaxanthins determination, respectively [21]. The betalains content (BLC) was calculated as:

(4) $$\mathrm{BLC}(\mathrm{mg}/L)=\frac{\left(A\times \mathrm{DF}\times \mathrm{MW}\times 1000\right)}{\left(\varepsilon \times 1\right)}$$

where A is the absorption value, DF is the dilution factor and 1 is the path length (1 cm) of the cuvette. For the quantification of Bc and Bx, the molecular weights (MW) and molar extinction coefficients (ε) were respectively, 550 g/mol and 60,000 L/(mol × cm) in H_2_O at λ = 538 nm for betanin, 339 g/mol and 48,000 L/(mol × cm) in H_2_O at λ = 480 nm for vulgaxanthin I.

### 2.9. Identification and Quantification of Phenolic Acids and Flavonoids

Chromatograms were recorded by RP-HPLC-UV/Vis (Shimadzu, Kyoto, Japan) using 280 nm for hydroxybenzoic acids, catechin, and epicatechin, 320 nm for hydroxycinnamic acids, and 360 nm for flavonoids. Separation was performed on a Luna C-18 RP column, 5 mm, 250 × 4.6 mm with a C18 guard column, 4 × 30 mm (both from Phenomenex, Torrance, CA, USA). Two mobile phases, A (acetonitrile) and B (1% formic acid) were used at flow rates of 1 mL/min with the following gradient profile: 0–10 min from 10 to 25% B; 10–20 min linear rise up to 60% B, and from 20 min to 30 min linear rise up to 70% B, followed by 10 min reverse to initial 10% B with additional 5 min of equilibration time [22].

### 2.10. Anti-Inflammatory (AIA) and Anti-Hyperglycemic Activity (AHgA) in Digested BPF

The anti-inflammatory activity was determined by protein denaturation bioassay using egg albumin (from a fresh hen’s egg) [23]. Briefly, 2 mL of the sample was incubated with 0.2 mL of egg albumin and buffered saline phosphate (pH 6.4) at 37 °C for 15 min and then at 70 °C for 5 min. After cooling, the absorbance was measured at 660 nm.

To examine in vitro anti-hyperglycemic activity α-glycosidase inhibitory potential was performed [24]. Each well contained 100 µL of 2 mmol/L 4-nitrophenyl α-D-glucopyranoside in 10 mmol/L potassium phosphate buffer (pH 7.0) and 20 µL of the samples at a concentration of 250 mg/mL, diluted in a buffer. The reaction was started with the addition of 100 µL of the enzyme solution (56.7 mU/mL). The plates were incubated at 37 °C for 10 min. The absorbance of 4-nitrophenol released from 4-nitrophenyl α-D-glucopyranoside was measured at 405 nm.

### 2.11. In Vivo Determination of Food Efficiency Ratio of Diet Supplemented with BPF

Eight-week-old male C57BL/6J mice, with initial body weights of 18–22 g, were randomly distributed among four groups (*n* = 8 mice/group) and housed in standard cages in a room with a 12 h light–dark cycle at a temperature of 22 ± 3 °C. The study was performed according to the regulations and standards of the Serbian Law on Experimental Animal Treatment and European Directive 2010/63/EU [25] and approved by the Ethics Committee for experimental animals welfare of the Faculty of Agriculture, University of Belgrade and Ministry of Agriculture, Forestry and Water Management (Process 323-07-00617/2017-05). Four groups were exposed to a standard pellet rodent diet without (CBD) and with the addition of 10 mg BPF per day (CBD + BPF) for 150 days and a high-fat (20% lard) and sucrose (20% sucrose as drinking solution) diet without (high-fat and sucrose diet (CBD*)) and with the addition of 10 mg BPF per day (CBD* + BPF). Before pelleting, 5 g of BPF was incorporated per kg of standard rodent food (Veterinary Institute Subotica, Serbia) based on corn, wheat, barley, and soybean protein. The pellet size was about 2 cm in length and 2 g in weight. Standard rodent food with and without 6.25 g BPF per kg was mixed well with pork lard in an 80:20 ratio to be used in the CBD* diet without and with 5 g BPF/kg. The average daily intake (2 g per mouse) contained approximately 10 mg of BPF. The calorie content of each diet was calculated using the following specific energy factors: 9 for fat, 4 for carbohydrates and protein, and 2 for dietary fiber. All groups had access to food and water *ad libitum*. The activity, behavior, and general health of the mice were monitored daily while food and water (CBD) or sucrose solution (CBD*) intakes and body weight were recorded weekly. The glycemic status was monitored throughout the experiment period, on a monthly basis. Fasting glucose was measured in fresh blood collected from the tail vein (one drop of full blood ~20 µL) on a biochemical analyzer—the A15 automatic analyzer (BioSystems S.A., Barcelona, Spain)—with standard glucose determination reagents (phosphate 100 mmol/L, phenol 5 mmol/L, glucose oxidase >10 U/mL, peroxidase >1 U/mL, 4-aminoantipyrine 0.4 mmol/L, pH 7.5) using glucose oxidase/peroxidase method. Results were expressed in mmol/L. An oral glucose tolerance test (OGTT) was performed within the last week of the diet. Overnight-fasted mice received a glucose solution (2 g/kg) by gavage. Blood was collected from the tail vein (one drop of full blood ~20 μL). Glucose concentration was determined prior and 30, 60, 90, and 120 min after glucose administration. Results were expressed in mmol/L. The area under the curve (AUC) was calculated geometrically as the sum of the areas of the trapezoids over 2 h including the area below the initial fasting glucose concentration.

After completion of the experiment the animals were euthanized and a macroscopic examination of tissues and organs was performed. The liver, kidneys, and heart were isolated. Organs were subjected to visual inspection and measured. The quantity of visceral fat was evaluated macroscopically.

### 2.12. Cookies Enrichment with BPF

The control whole spelt flour (SF) cookies were prepared as described by Mitrevski et al. [26]. The cookies with 20% BPF were prepared by replacing part of SF with BPF in the formulation for the control sample. SF (90 g) was mixed with BPF (60 g), as well as softened extra virgin coconut oil (60 g), cane sugar (50 g), water (40 g), and baking powder, and after the mixture rested for 30 min the cookies were cut using a round mold with a diameter of 4.7 cm, baked for 12 min at 150 °C, allowed to cool for two hours, and stored without exposure to light before analysis. Proximate composition was determined and energy value was calculated as described above.

### 2.13. In Vivo Determination of Glycemic Index

The study included 10 non-diabetic adults aged 22–59 years old (6 females and 4 males), with body mass index (BMI) within the range of 19–23 kg/m^2^ and fasting glucose level ≤ 6 mmol/L (SRPS ISO 26642:2013) [27]. Informed consent was obtained from all of them. The Ethics Committee of the Institute of Public Health of RS approved the study (No of ethics approval 5646/1 issued 7 October 2022). One test was carried out per week. The participants fasted for at least 12 h and avoided exercise, smoking, alcohol, and drug consumption. Blood was taken 5 min before consumption of samples (0 min). Fasting glucose tests were performed using 250 mL of water containing 25 g of glucose, or an equivalent amount of carbohydrates (25 g) provided by cookies, which were consumed within 5 min. Subsequent blood glucose tests were carried out 15, 30, 45, 60, 90, and 120 min after sample consumption. Area under the curve (IAUC) was calculated geometrically as the sum of the areas of the trapezoids over 2 h excluding the area below the initial fasting glucose concentration. The glycemic index (GI) was calculated by averaging the IAUC for each cookie, dividing it by the IAUC for glucose, and multiplying by 100. The glycemic load was calculated by multiplying GI by the weight in grams of the available carbohydrates in the cookies and then dividing by 100 (SRPS ISO 26642:2013) [27]. Significant differences were determined at *p* < 0.05.

### 2.14. Determination of Textural and Sensorial Properties of Enriched Cookies

The hardness of cookies was determined by diametrical compression on a TA-HDPlus Texture Analyzer (Stable Micro Systems, Surrey, UK) using a 30 kg load cell and a P/2 (steel, 2 mm diameter) probe. The instrument setting parameters during the test were as follows: speed before measurement—2 mm/s, speed during measurement—3 mm/s, speed after measurement—10 mm/s, and trigger force—25 g. Based on the obtained data, hardness was calculated as the highest recorded force until break. Elasticity was calculated as the travel distance of the probe until first break. Functional products enriched with BPF and control were tested in parallel. Testing was conducted triplicate at 4 °C. All measurements were performed in triplicate, and results were expressed as mean ± standard deviation. Differences between control and BPF-enriched cookies were evaluated using one-way analysis of variance.

Consumer testing was performed with 30 consumers affiliated with the University of Belgrade. Participants voluntarily took part in the study after providing informed consent. The functional product was evaluated for overall acceptance, texture acceptance, and flavor acceptance using a 9-point hedonic scale (1 = dislike extremely, 5 = neither like nor dislike, and 9 = like extremely), as described in previous report Zlatanovic at al. [28]. A consumer survey was conducted using a structured questionnaire. Demographic data, including age, gender, and diet regime (if any), were collected, followed by questions on dietary habits and attitudes toward functional foods, particularly sweets. The questionnaire addressed habits related to confectionery consumption and sugar intake, the perceived importance of high fiber content, and attitudes toward the reusage of food by-products in the food chain. Respondents were asked about their acceptance of products containing by-products commonly treated as waste in Serbia. Purchase intention was evaluated as a preliminary measure of market potential, reflecting consumers’ willingness to purchase the product. Responses were recorded and analyzed using descriptive statistics. The full questionnaire is provided as Supplementary Material.

### 2.15. Statistical Analysis

Statistical analysis of the data was conducted using the XLSTAT (version 2014.5.03, Addinsoft, New York, NY, USA), analysis and statistics add-in for MS Excel. All in vitro measurements were performed in triplicate. The results were expressed as means ± standard deviation (SD). Significant differences between means were determined by t-test and ANOVA with post hoc Tukey’s HSD (honest significant test) test (*p* < 0.05).

## 3. Results and Discussion

### 3.1. Stability and Safety of BPF

In the applied technological procedure [12] the wet beetroot pomace (BP) obtained by pressing thoroughly washed beetroot was collected aseptically from an industrial juice producer operating under HACCP principles and good manufacturing practice, and dehydrated immediately after juice production at approximately 55 °C under strict hygienic conditions to a moisture content of 3.4 wt% and a water activity (aw) of 0.2, and subsequently ground to a particle size of ≤300 µm. Good stability and storability of the obtained BPF was confirmed by monitoring aw during storage, which remained within the limits of safe microbiological stability and reached 0.34 after one year without any increase in moisture content. Despite a slight increase over time aw remained well below 0.6, supporting the microbiological stability of BPF.

In parallel, thermodynamic properties were assessed with an emphasis on the glass transition temperature (Tg) as an additional indicator of physical stability. The substantially high Tg (45.8 ± 2.0 °C) well above the common storage temperature (22 °C), was maintained after a year of storage (46.0 ± 1.1), indicating the good physical stability of the BPF. The consistently low aw and unchanged Tg over a year of storage confirm the physicochemical stability of BPF and a minimal risk of microbiological contamination [29]. Water activity strongly influences the glass transition temperature because water acts as a plasticizer, enhancing molecular mobility and reducing the Tg.

The safety of BPF was confirmed by the acute oral toxicity test conducted in accordance with OECD Guideline No. 423 [14]. Neither immediately after administration of BPF at a dose of 2 g/kg body weight via gastric gavage to experimental animals, nor over a 14-day observation period were signs of toxic reactions observed. While the acute oral toxicity assessment confirmed the absence of toxic effects of single-dose exposure, it should be emphasized that toxicological and microbiological safety represent complementary aspects of overall product safety. The reuse of plant-based side streams, such as BP, requires consideration of chemical and microbial hazards. While most studies focus on chemical hazard [30], typically more difficult to remove during further processing, microbiological risks should not be overlooked, particularly when side streams are intended for direct consumption. Thus, complementary microbiological analyses of BPF would be required, as the presence of soil-associated microorganisms in beetroot by-products cannot be fully excluded. The potential risk for human consumption largely depends on beetroot cultivation, storage, pretreatment, and processing conditions during juice production, as well as on the methods of stabilization and further storage of BPF. Previous studies reported that BP can be pretreated by soaking in a 200 ppm chlorine solution, followed by moisture removal and drying at 60–80 °C to obtain flour with a final moisture content of 10–12% [31]. Also, beetroots juice was microbiologically tested to verify safety [32]. However, the shelf life and thermal stability of the BP flour or powder was not the subject of previous studies. The dehydration process applied in this study at moderate temperatures (approx. 55 °C) is designed to preserve bioactive compounds and reduce moisture content and aw, rather than to ensure microbial inactivation. Thus, microbiological assessment of BPF is recommended even in bakery or confectionery applications where thermal processing offers an additional safety barrier, but it remains especially advisable when BPF is intended for direct consumption as a dietary supplement.

### 3.2. Proximate Composition

The proximate composition of BPF (Table 1) confirms low lipid content (0.62 ± 0.07 g/100 g), which is in agreement with data from the literature indicating that beetroot by-products are generally low in fat, contributing to their oxidative stability [30]. Protein content (12.0 ± 0.3 g/100 g) has been found to be similar to values reported for minimally processed BP (12–13%) [31].

During the pressing and juicing, a large amount of sugar from the beetroot was transferred to the juice. Thus, the resulting BP contains significantly lower sugar levels and higher dietary fiber content. However, BP from different juice producers often shows substantial variability, due to differences in processing technologies, raw material origin and agronomic conditions [4]. Although the dietary fiber content of BP can vary considerably depending on the starting material and, in particular, the juicing process, in this study BP obtained from a single industrial producer operating under standardized conditions has been used, resulting in batches with relatively limited variability in composition and physicochemical characteristics. The total dietary fiber content in BPF was 26.7 g/100 g, while reported values varied from ~20% [33] to 60% [34]. Even higher content of fiber was found in beetroot peels, parings, and stalks flour (~65%) [31]. All obtained values were notably higher than those reported in the literature for whole beetroot powder [26], as well as those declared for commercially available beetroot powder products [35].

Similarly, the sugar content of beetroot pomace flour (BPF) obtained in this study (50.6 ± 0.8 g/100 g) was lower than the values reported in the literature [26] or declared for commercially available beetroot powder, which can be attributed to the removal of a substantial portion of soluble sugars during juice extraction. The observed predominance of sucrose is in line with the sugar profile of beetroot and its by-products, where sucrose is typically the major component. Both beetroot juice and BP have been reported to contain significant levels of sucrose, glucose, and fructose, although the exact values depend on moisture content and processing [36].

Overall, BP exhibits a nutritional profile consistent with previous studies, characterized by high protein and carbohydrate contents, low fat levels, and a notable dietary fiber fraction.

### 3.3. Effect of BPF in Flour Fortification

Composite flours may represent a strategy for food security and a promising alternative to traditional flour [37]. Their functional properties, diverse applications in food production, and potential to improve food security are highlighted along with challenges related to ingredient compatibility, processing optimization, and consumer acceptance [38]. Until now, the utilization of pomace flour in composite flour formulation, or flour fortification has not been fully addressed. Thus, providing a comprehensive perspective on the utilization of pomace flour such as BPF in composite flour might be of interest.

Here, the carbohydrate:fiber ratios of BPF and cereal and pseudo-cereal flour were compared to evaluate BPF potential in flour fortification and composite flour development. Total DF content in BPF (~27 g/100 g) was found to be much higher than in wheat and wholewheat flour (3.45 ± 0.01 and 11.4 ± 1.3 g/100 g) as well as gluten-free flours from rice (0.43 ± 0.15 g/100 g), buckwheat (2.18 ± 0.11 g/100 g), oat (4.05 ± 0.40 g/100 g) and maize (2.62 ± 0.45 g/100 g) [39]. Moreover, the significant content of fructans indicated a prebiotic effect, while in contrast to cereals BPF does not contain anti-nutritive factors such as phytic acid or allergens such as gluten. Comparison between the ratio of total carbohydrates to total DF in BPF (1.9) and rice (181), buckwheat (29), maize (28), wheat (21), oat (18), sorghum (18), spelt (17), quinoa (8) and whole wheat (6), reveals the potential of BPF to contribute to the content of DF in bakery and confectionery products without overloading it with energy.

**Figure 1:**
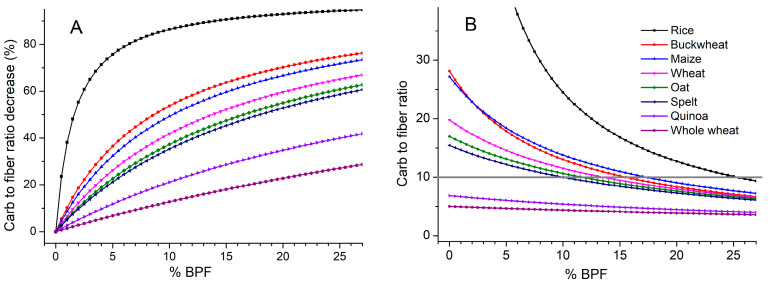
(**A**) Carb:fiber ratio decrease (%) of cereal and pseudocereal flour (rice, buckwheat, maize, wheat, oat, spelt, quinoa and whole wheat flour) with an increase in the percentage of BPF added and (**B**) BPF content (%) required to reach 10:1 ratio.

Figure 1A clearly demonstrates that even a 5% addition of BPF in gluten-free rice flour, a staple food in much of the world and a key component of the gluten-free diet, significantly alters the carb-to-fiber ratio (by 80%). Refined rice flour is the most commonly used in many gluten-free formulations, followed by maize flour and refined starches with negligible fiber content (<1 g/100 g) [40]. However, the fortifying potential of BPF is not limited to refined gluten-free flours; its efficacy in enhancing standard types of flours is also evident (Figure 1A). Even in whole-grain or pseudocereal flours already rich in fiber, a decrease in the carb-to-fiber ratio was observable, as seen in whole wheat and quinoa flour. The simple mathematical approach applied here might be useful in optimization of composite flour content. As seen in Figure 1B, from 14 to 28% of BPF has been required to be added to spelt, sorghum, oat, wheat, buckwheat, maize, and rice flour to reach the recommended carb:fiber ratio (10:1). This approach could facilitate the provision of fortified flour as a staple food with increased fiber content, thereby contributing to public health by helping prevent NCD associated with inadequate fiber intake. It is worth noting that BPF also contributes a variety of bioactive compounds, including polyphenols tightly bound to fiber, which are released in the colon through the action of the gut microbiota, providing additional health benefits [41]. The impact of different processing techniques, including baking, frying, roasting, or extrusion, on the retention and bioavailability of essential nutrients in products from flour enriched with BPF remain to be comprehensively studied. Also, the effects of storage conditions, moisture management, packaging and shelf life need to be studied.

### 3.4. Polyphenolics Compounds of BPF

Besides high fiber content, a significant amount of phenolics contributed anti-obesity effect of BPF. Bioactive molecules from fruit and vegetable waste are known to protect against endothelial damage that causes obesity-related complications such as hypertension, dyslipidemia, diabetes, and metabolic syndrome [42]. The association between polyphenols—known as modulators of the physiological and molecular pathways involved in energy metabolism—acting in the stimulation of β-oxidation and adipocyte differentiation inhibition, and counteracting oxidative stress and obesity was found to be dependent on the amount consumed and their bioavailability [43].

Total phenolic content (TPC) in BPF digested in simulated gastric and intestinal conditions (14.1 ± 0.1 mg GAE/g) was considerably higher than in apple pomace flour (6.5 ± 0.1 mg GAE/g) [10] and markedly higher than values reported for conventional cereal flour such as wheat (0.13 ± 0.02 mg/g), rice (1.42 ± 0.24 mg/g) and maize (0.98 ± 0.10 mg/g), or pseudocereal such as buckwheat (4.66 ± 0.22 mg/g) [39]. The determined TPC value falls within or slightly above the range reported for beetroot pomace (BP) extracts (1.9–12.0 mg GAE/g dry weight) obtained from different beetroot varieties (‘Cardeal-F1’, ‘Egyptian’, ‘Bicor’, and ‘Kestrel’) [35], and approaches the upper values reported for various parts of beetroot (4.2–15.5 mg GAE/g dry weight) [44]. Some studies reported substantially higher values, up to 45.7 mg GAE/g, depending on extraction conditions and beetroot variety [45]. The phenolic content of BP extracts strongly varies with the solvent showing lowest values for water and the highest for ethanol extract [46,47]. Compared with conventional solvent extraction, in vitro digestion is more relevant from the perspective of potential biological effects, as the TPC values measured in the digested samples may better reflect the bioaccessible compounds and their potential effects in vivo.

The content of flavonoids (TFC), expressed in mg of quercetin equivalents per g (1.43 ± 0.10 mg QE/g) confirmed previous findings that TFC represent a smaller share (~5%) of the polyphenols present in the pomace [48]. The content of total flavonoids in encapsulated BP was 10.2 ± 1.2 mg rutin equivalent/100 g dry weight. Similar to TPC, TFC depended on the extraction method, beetroot variety and processing.

Individual phenolic compounds identified in digested BPF by RP-HPLC-UV/V show that among flavonoids epicatechin (11.9 ± 0.4 mg/100 g) was the predominant, followed by catechin (3.1 ± 0.1 mg/100 g). Among the phenolic acids, vanillic (77.7 ± 3.6 mg/100 g) was the most abundant, followed by protocatechuic (51.9 ± 2.3 mg/100 g), gentisic (23.9 ± 1.0 mg/100 g) and p-hydroxy benzoic (5.2 ± 0.2 mg/100 g), with smaller amounts of p-hydroxybenzoic, chlorogenic, and gallic acids. The total content of all determined phenolic compounds was 183 mg/100 g. Previous reports demonstrated that p-hydroxy benzoic acid was the prevalent among the phenolic acids in beetroot extracts, followed by cinnamic, vanillic, chlorogenic, trans-ferulic, and caffeic acid [49]. Furthermore, HPLC analysis of Detroit BP extract revealed a different distribution pattern, with ferulic acid (132.5 mg/100 g) and catechin (38.0 mg/100 g) as major constituents, while vanillic and p-hydroxybenzoic acids were present in considerably lower amounts [4]. These differences in phenolic profiles can be attributed to varietal differences, extraction procedures, etc. Compared to conventional solvent extracts the application of in vitro digestion may have altered the release and relative abundance of specific compounds.

The content of betacyanins and betaxanthins in digested BPF (898 ± 54 and 960 ± 65 µg/g) is consistent with previous observations indicating no significant difference in their content in beetroot extracts (*p* > 0.05) [48]. Variations in betacyanin (18.8–24.2 mg/g dry extract) and betaxanthin (11.2–22.9 mg/g dry extract) contents were smaller than those observed for TPC and TFC [46]. Although direct comparison is limited due to the lack of data for digested samples, the obtained result is still within the range of previously reported values for BP extracts. Vulić et al. reported that the betalain content in BP ranged from 0.75 mg/g dry weight for cv. ‘Egyptian’ to 3.75 mg/g dry weight for cv. ‘Bicor’ [50], confirming that significant amount of betalains remain bioaccessible after simulated digestion.

The AO activity of the BPF digest determined by the FRAP and DPPH assays was high (31.5 ± 1.1 and 25.8 ± 2.9 µmol TE/g, respectively), as expected based on the high content of TPC, TFC and betalain. The DPPH-free radical scavenging activity of BP extracts, reported previously, expressed as EC_50_, ranged from 0.133 mg/mL to 0.275 mg/mL [46]. A moderate to strong correlation between AO activity of BP extract and phenolic compounds indicated that phenolic acids and flavonoids are major contributors to radical scavenging capacity [51]. A positive relationship between the antiradical activities of BP and betaxanthins was also shown [46]. The AO activity of BP extracts from different beetroot varieties evaluated using multiple AO assays, revealed significant variation depending on the cultivar and indicated that phenolic compounds and betalains are the main contributors to AO activity [51]. Generally, reported values of AO activity tend to be higher in pomace extracts compared with raw beetroot samples.

Importantly, most studies on beetroot or BP extracts report stability over relatively short storage periods (typically up to two months), including encapsulated systems designed to improve phenolic preservation [48,52]. However, there are currently no studies addressing the thermal stability and long-term shelf-life of BPF itself. In the present study, BPF demonstrated remarkable stability over one year of storage, indicating that the applied technological process effectively preserves phenolic compounds and maintains antioxidant (AO) activity. A good retention of phenolics and AO activity was observed, with TPC and FRAP decreasing by less than 5%, while DPPH scavenging activity decreased by 15% but remained at a high level. This stability may be attributed to the intrinsic BPF matrix, which can provide better protection of phenolic compounds than isolated extracts or encapsulated systems, where bioactive compounds are removed from their natural matrix and may therefore show lower retention during storage. While the majority of published studies focus primarily on free or encapsulated BP extracts, the approach adopted in this study provides complementary insight into the behavior of bioactive compounds within the intrinsic whole food matrix subjected to simulated in vitro digestion. From a functional food perspective, maintaining phenolics within their original matrix may represent a more sustainable alternative, particularly in the context of whole agri-food by-product valorization. These results therefore suggest that BPF should not be viewed merely as a source of extractable phenolics, but as a stable food matrix with superior stability, bioaccessibility, and potential physiological functionality.

### 3.5. In Vitro Determination of Antihyperglycemic and Anti-Inflammatory Activity of BPF

Antihyperglycemic (AhgA (%)) and anti-inflammatory activity (AIA (%)) was determined upon in vitro digestion of BPF. By centrifugation of digested BPF (1 g) 10.0 mL of supernatant was obtained. Significant AhgA (27.3 ± 1.3%) and AIA (41.0 ± 3.4%) were ascribed to BPF compared to diclofenac sodium at a concentration of 0.25 mg/mL (47.7 ± 2.3%) and acarbose at a concentration of 0.5 µg/mL (35.4 ± 1.5%) as standards, respectively. Previous studies addressing the effect of beetroot supplementation on inflammation, oxidative stress, cognition, and endothelial function also indicated its potential in the treatment of a range of clinical pathologies associated with oxidative stress and inflammation [53]. The antioxidant, anti-inflammatory, and chemo-preventive activity of betalain was shown both in vitro and in vivo [53]. The betalains have anti-inflammatory properties as well as high AO activity. In addition, highly active phenolics such as caffeic acid, epicatechin, and rutin are known as the organic antioxidants [54]. Considering that supplementation of foods with anti-inflammatory potential represents an approach to mitigate obesity-induced inflammation [55], the results obtained in this study might be relevant in the context of the potential contribution of BPF to obesity prevention.

### 3.6. Effect of Long-Term BPF Diet Supplementation

Within the scope of this animal study, the long-term (150 days) effects of an addition of 10 mg of BPF per day (0.5% *w*/*w*) to a standard cereal-based diet (CBD) and high-fat and sucrose diet (CBD*) on glycemic status, body weight gain (BWG) and glucose metabolism in oral glucose tolerance test (OGTT) were followed. Results obtained for CBD-BPF and CBD*-BPF groups were compared with CBD and CBD* without the addition of BPF. Fasting glycemia, BWG, daily intake of food, water, and energy are shown in Table 2.

Previously, the dehydrated beetroot stalk and leaves were reported to decrease the fasting glycemia and enhanced insulin resistance in rats exposed to a high-fat diet [56]. Clinical studies highlighted that beetroot products maintain blood glucose levels in humans with type 2 diabetes and obesity but differences in studies design, dosage, duration, etc., caused inconsistencies [7]. In this study, in addition to fasting glucose levels, feed efficiency ratio was also assessed, providing complementary insight into the effects of BPF on BW regulation. Feed efficiency is a particularly important parameter, as it reflects how effectively consumed energy is converted into body mass. Moreover, feed efficiency is widely reported in dietary intervention studies using fiber or polyphenol rich food, or agro-industrial by-products, enabling direct comparison with previously published data.

The food efficiency ratio calculated as BWG per food intake—food efficiency ratio 1 (FER1) and per energy unit—food efficiency ratio 2 (FER2), and the area under the OGTT curve, are shown as well. The CBD* group showed a statistically significant (*p* < 0.05) increase in glycemia (39.3%) in comparison to CBD, while BWG was not statistically different. The CBD*-BPF group showed no increase in glycemia compared to CBD. Glycemia in CBD*-BPF decreased by 38.7% compared to the CBD* group. In CBD*-BPF and CBD-BPF, BWG was significantly suppressed in comparison to diets without BPF (60.7% and 39.2%, respectively). BWG and FER1 significantly differ between diets with and without BPF.

**Figure 2:**
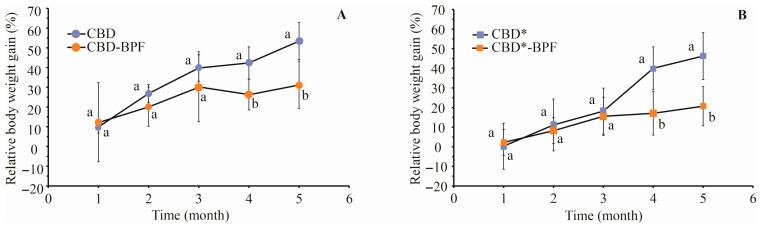
Effect of (**A**) CBD and (**B**) CBD* with and without beetroot pomace flour (BPF) addition on relative body weight gain of mice during 150 days of exposure. Different lowercase letters within the same time indicate a significant difference in means, according to t-test (*p* < 0.05) (*n* = 8); the asterisk (*) denotes groups exposed to a high-fat and sucrose diet, with or without BPF CBD—cereal-based diet; CBD-BPF—CBD supplemented with 10 mg of BPF; CBD*—high-fat and sucrose cereal diet; CBD*-BPF—high-fat and sucrose cereal diet with 10 mg of BPF).

A statistically significant difference (*p* < 0.05) was observed in daily food intake between the CBD* and CBD groups although they were close. However, energy intake was more than 2.5 times higher in the CBD* group. FER1 and FER 2 were lower in CBD*-BPF and CBD-BPF than in groups without BPF. The difference in FER1 between CBD* and CBD as well as CBD*-BPF and CBD-BPF was not found statistically significant. However, FER1 of CBD*-BPF and CBD-BPF were reduced by 2.6 and 1.7 times compared to CBD* and CBD, respectively. A similar reduction in FER 2 was noticed. An insight into the impact of long-term BPF supplementation on weight management BWG was followed monthly (Figure 2).

Differences in BWG in the CBD*-BPF and CBD-BPF groups in comparison to the CBD* and CBD groups became more prominent at the end of considered period. Due to the lower food intake at the beginning of exposure, the BWG increase in the CBD* group was lower but almost caught up with the CBD group. At the end of the period considered, no significant difference in BWG between the CBD and CBD* groups were noticeable. By far the lowest BWG was noticed in the CBD*-BPF group during the entire period considered. Significant effects of BPF on BWG reduction were observed in the CBD-BPF group, in comparison to the CBD group.

Although the concepts of animal feed studies differ from experimental design applied here, the results are comparable, showing reduced feed utilization and growth performance in relation with increased intake of fiber from plant sources or agro-industrial by-products. High levels of soluble and insoluble fiber in pig and broiler diets impaired nutrient digestion, altered gut microbiota, and decreased feed efficiency [57,58]. Evidence that fiber originated from fruit and vegetable by-products lowers feed conversion efficiency (a reciprocal measure of feed utilization compared to feed efficiency) is consistent with our finding that mice fed BPF gained less BW, likely due to reduced energy utilization. In humans, increased intake of fruit and vegetable fiber has been associated with improved BW regulation and glycemic control. Randomized controlled trials and meta-analyses indicate that higher fiber intake reduces fasting glucose, insulin, and BW in overweight and obese adults [59,60], supporting the relevance of employing by-products such as BPF in dietary strategies for BW management and glucose regulation.

In addition to fiber, beetroot bioactive compounds, particularly polyphenols and betalains, may contribute to BW regulation. Previous studies demonstrated that beetroot juice decreased BWG in obese rats [61]. Mechanistic investigations suggest that this effect may be mediated, at least in part, by activation of brown adipose tissue through increased UCP1 gene expression, promoting thermogenesis and energy expenditure. These findings indicate that the modulatory effects of beetroot polyphenols on metabolism can complement fiber-mediated mechanisms, contributing to improved energy balance and reduced BWG, as observed in this study in animal groups on BPF supplementation.

Although elucidating the underlying mechanisms of action was not the aim of this study, the prevention of BWG observed may be attributed to a significant reduction in energy intake, a well-established effect of fiber-rich foods, as well as to the modulatory effects of polyphenols on carbohydrate metabolism, as evidenced by the notable anti-glycemic activity observed. Both physical (fiber-related) and biochemical (polyphenol-mediated) mechanisms may therefore contribute to the observed outcomes. Gut microbial populations were not assessed but the combination of fiber and phenolic compounds in BPF may modulate microbiota composition, influencing nutrient utilization. Having in mind the considerable implications of obesity prevention for personal and national health, BPF produced at an industrial scale level by the application of already developed technology [12] can be considered as natural product with potential to prevent obesity. It can be used for the enrichment of various food products or as dietary supplement.

**Figure 3:**
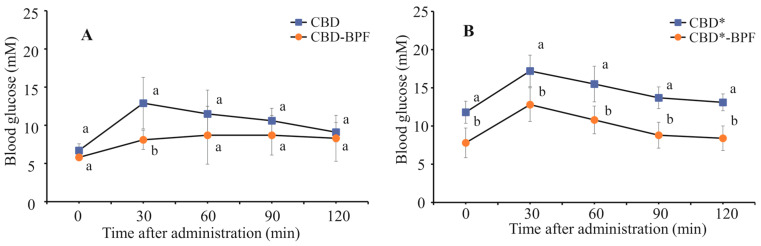
Effect of 150 days exposure to (**A**) CBD and (**B**) CBD* with and without beetroot pomace flour (BPF) addition on plasma glucose concentrations in OGTT in mice. Different lowercase letters within the same time indicate a significant difference in means, according to the t-test (*p* < 0.05) (*n* = 8); the asterisk (*) denotes groups exposed to a high-fat and sucrose diet, with or without BPF CBD—cereal-based diet; CBD-BPF—CBD supplemented with 10 mg of BPF; CBD*—high-fat and sucrose cereal diet; CBD*-BPF—high-fat and sucrose cereal diet with 10 mg of BPF).

In addition to fasting glucose levels and feed efficiency, an additional parameter related to glucose homeostasis was assessed to provide further insight into the metabolic effects of BPF. The influence of BPF on glucose metabolism in OGTT was investigated as well. As it is highly sensitive and specific in detecting glucose intolerance, OGTT was performed within the last week of the diet. Improved glucose metabolism in the OGTT (Figure 3) confirmed that BPF supplementation in the amount of 0.5% *w*/*w* led to better glucose tolerance in animals exposed to CBD*.

The values of OGTT AUC for CBD* (29.0 ± 3.6 mMh) and CBD*-BPF (19.2 ± 2.0 mMh) were significantly different. Their ratio of 1.5 indicated a significant improvement in glucose tolerance in CBD*-BPF. During the entire glucose challenge, the difference between CBD and CBD-BPF was observable but not statistically significant, as was the difference between AUC values (19.9 ± 4.5 and 16.3 ± 3.7 mMh). These results suggest that BPF supplementation can modulate glucose metabolism, probably by the combined effects of fiber and polyphenols, which slow carbohydrate absorption and improve glycemic regulation.

A macroscopic examination of tissues and organs revealed the lower quantity of visceral fat in groups exposed to BPF and no visible changes in organ appearance, structure, or size, with weights as follows: liver 1.45 ± 0.07 g, kidney 0.48 ± 0.05 g, and heart 0.18 ± 0.02 g. Similarly, no statistically significant differences were observed in serum lipids (total cholesterol or triglycerides) between the groups. However, adding dehydrated beetroot stalk and leaves to rats diminished the deteriorating effect of the high-fat diet by reducing total cholesterol and LDL [62]. Abdo et al. [61] previously reported that a beverage enriched with beetroot leaf and stem juice prevented obesity in a high-fat diet rat model, attenuating oxidative stress by restoring catalase and glutathione peroxidase activities and by improving liver enzymes and lipid profiles. Together with the present findings, this indicates that various beetroot by-products can help regulate BW. To further elucidate the underlying mechanisms of BPF action, additional studies that include measurements of insulin levels and sensitivity, adipose tissue distribution and inflammatory markers, as well as analysis of gut microbiota and short-chain fatty acid production, are required.

### 3.7. Effect of BPF Incorporation in the Functional Product on Glycemic Response

Cookies were chosen to be enriched with BPF due to their popularity and wide consumption. To highlight the effect of BPF enrichment in cereal flour that already has a carb:fiber ratio close to 10:1, whole spelt flour was chosen. At a pilot scale level, whole-spelt cookies and cookies with 20% of spelt replaced with BPF were produced in parallel. Cookies with BPF contained 24.0 ± 0.7 of fat, 50.7 ± 2.0 of available carbohydrate, 26.6 ± 1.0 of total sugar expressed as a sum of glucose (0.20 ± 0.03), fructose (0.16 ± 0.03), and sucrose (26.2 ± 0.9), 9.9 ± 0.6 of protein, 7.9 ± 0.6 of a total fiber expressed as a sum of insoluble fiber (5.1 ± 0.2) and soluble fiber (2.0 ± 0.2), plus 0.80 ± 0.11 of fructan expressed as g/100 g. The energy value of cookies was 474 ± 2 Kcal (1979 ± 9 KJ) per 100 g (Table 3).

The enrichment of grain-based products with BP was already related to an increase in DF content [63]. Wheat flour was substituted with BP at various levels, up to 50%. Cakes containing 20% BP received the highest acceptability ratings. However, the effect of BP addition on carb:fiber ratio and postprandial glucose was not discussed [26].

**Figure 4:**
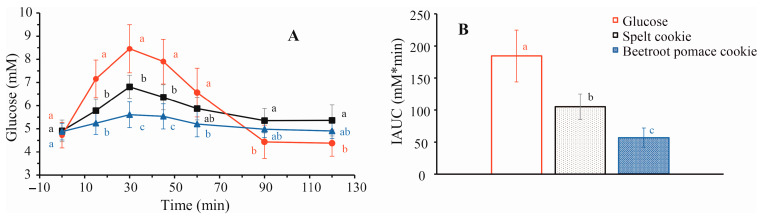
(**A**) Glycemic curve and (**B**) IAUC after consumption of 25 g of glucose (●), spelt flour cookies (■) and cookies with 20% BPF (▲) containing the same content (25 g) of available carbohydrates. Different lowercase letters indicate a significant difference in means, according to Tukey test (*p* < 0.05) (*n* = 10).

Here, the calculated carb:fiber ratio (6.4:1) of BPF cookies was in accordance with American Diabetes Association ADA recommendations for cereal products while control cookies were well above it (17.8:1). Postprandial glucose after consumption of spelt cookies, cookies with 20% BPF, and pure glucose was compared. The effect of BPF was noticed both in decreasing the percentage of maximum rise in plasma glucose and in a reduction in the area under the glucose levels (IAUC). The glycemic curve was steadier in the cookies’ groups than in the glucose group (Figure 4). The curve and area under the glycemic curve (IAUC) demonstrated a significantly improved glucose of both cookie groups in comparison to the glucose group. Statistically significant differences were also observed between cookie groups (*p* < 0.05) confirming that the addition of BPF lowers glucose response. Based on calculated GI, BPF cookies were classified as low-GI types of food (30 ± 11) while spelt cookies belong to media-GI (56 ± 17). It was also found that BPF cookies belong to a low-GL food (GL < 10). Such data allowed for a much higher intake allowance of BPF cookies than control ones. Values in the literature for cookies are commonly about 70 [64] while low GI (42) was ascribed to fiber-enriched cookies [65]. Fiber causes negligible direct blood glucose response [66]. The percentage of fiber corresponds with slower digestion and a lower increase in glucose since fiber participates in the mechanism responsible for the glucose retardation effect that may markedly reduce the access of glucose to the epithelium. Therefore, this product aligns with the dietary guidelines for diabetics [67], which emphasize the consumption of foods rich in dietary fiber and with a low glycemic index. Although preliminary, these findings offer a promising foundation for the development of functional products incorporating BPF.

### 3.8. Estimation of Textural and Sensorial Properties of Functional Product

In terms of texture, the control sample exhibited higher, but not significant hardness (7138 ± 1038 g) compared to the sample enriched with BPF (6002 ± 1098 g), indicating a firmer and more compact structure. Conversely, the BPF-enriched cookies demonstrated significantly greater elasticity and work (1.68 ± 0.19 mm and 4754 ± 924 mJ, respectively) in comparison to the control (0.92 ± 0.02 mm and 1770 ± 250 mJ, respectively), suggesting improved flexibility and energy absorption during deformation (Table 4).

Specifically, while control cookies presented higher hardness values, the difference was not statistically significant, corroborated by studies indicating a lack of substantial hardness variation following dietary fiber integration into bakery products [68]. Studies have suggested that insoluble dietary fibers dilute the starch–protein matrix of cookies without leading to significant increases in overall firmness [69]. This can be attributed to disruptions in the structural network formed during the baking process due to the partial replacement of spelt flour with BPF, which limits the formation of a cohesive matrix [70,71]. Elasticity was notably improved in BPF-enriched cookies, contrasting with the lower elasticity values associated with control cookies, typical of more brittle cookie structures. The significant increase in elasticity for BPF cookies indicates a shift toward enhanced deformability and elastic recovery [71]. This alteration in mechanical properties is consistent with findings from studies on bakery products fortified with fruit or vegetable pomaces, attributing observed improvements to the high water-binding capacity of beetroot pomace fiber [70]. Furthermore, the incorporation of BPF resulted in structural modifications, as evidenced by the work of compression. Control cookies required less energy to deform, demonstrating brittleness, while BPF-enriched cookies absorbed significantly more energy before fracturing [72,73]. This finding aligns with previous research suggesting that fiber particles enhance energy dissipation during mechanical stress, thereby fostering a tougher internal structure [69,71]. The cumulative effects of BPF inclusion indicate a transition from a brittle to a more ductile structure, characterized by enhanced energy absorption and microstructural hydration. Although hardness did not show a statistically significant change, the prominent increases in elasticity and the work of compression affirm the substantial impact of dietary fiber on the textural response of baked products [73,74]. The findings are corroborated by studies on cookies enriched with various pomace types, such as apple and grape, suggesting that such ingredients serve as effective fillers within the cookie matrix, promoting cohesive properties and reducing crack propagation during mechanical stress [75,76].

In summary, despite a non-significant impact on hardness, the integration of BPF into whole spelt flour cookies significantly influenced elasticity and the work of compression, illustrating the technological potential of BPF as a functional ingredient. These results not only enhance the nutritional profile of the cookies but also their textural resilience, signaling beneficial changes within the dough matrix attributed to fiber incorporation [72,77].

The changes observed in cookie hardness, elasticity, and work of compression reinforce the notion that BPF alters the mechanical responses of spelt flour cookies. The results advocate for further exploration of dietary fiber in bakery formulations, confirming the role of BPF as a valuable functional ingredient in enhancing both the nutritional and textural quality of baked goods.

The taste and odor of cookies enriched with BPF was found acceptable by consumers. Looking at the total number of respondents, ‘overall acceptance’, ‘texture acceptance’ and ‘flavor acceptance’ were scored with the average hedonic scores: 7.0 ± 0.9, 6.8 ± 1.0 and 6.7 ± 0.8, respectively, indicating that the tested respondents liked the product. The results of hedonic analysis provided [28] an insight into consumer perception as a preliminary step towards evaluation of the commercial potential of the enriched cookie. The consumer survey, which assessed both sensory preferences and appreciation of nutritional attributes, indicated that respondents valued the high fiber content, and the great majority expressed a positive purchase intention. These preliminary findings are in accordance with previous studies reporting acceptable sensory characteristics of cookies enriched with beetroot by-products, while simultaneously offering improved nutritional profiles, such as high fiber content [78].

Both textural and sensorial testing indicated the feasibility of incorporating BPF into confectionery products such as cookies. The results highlight the potential of BPF as sustainable, fiber-rich functional ingredient, supporting nutritional enhancement and agro-waste valorization. In this way, high-value by-products would contribute wellbeing, circularity in the fruit and vegetable processing industry, and prevent environmental pollution caused by inadequate treatment of wet pomace, prone to spoilage and the emission of greenhouse gases.

Although the utilization of BP as a functional ingredient may contribute to reducing food waste, scaling up the dehydration process would require drying and transport, increasing energy consumption and environmental impact. Therefore, a comprehensive evaluation, including a full life cycle assessment (LCA) and a cost–benefit analysis (CBA), is required in future studies to fully assess the economic viability and sustainability of suggested valorization strategy.

## 4. Conclusions

The valorization routes for industrially produced BPF were proposed on the basis of comprehensive physicochemical and techno-functional characterization, demonstrating its suitability as a functional ingredient with potential to contribute to reducing the dietary fiber gap in the modern diet. The study uniquely evaluated the carbohydrate-to-fiber ratio of BPF relative to commonly consumed cereal and pseudo-cereal flour, including calculation of the amount needed to reach the recommended 10:1 ratio, as a practical, application-oriented nutritional benchmark for cereal-based formulations. A significant decrease in carb:fiber ratio in relation to BPF content in mixtures with various grain flour indicated that BPF addition into the cereal-based diet or incorporation into cereal-based confectionery could impact nutritional profile. The functional applicability of BPF was validated at pilot scale through incorporation into a popular confectionery product, resulting in a corresponding reduction in the carb-to-fiber ratio as well as glycemic response determined in a human trial. Furthermore, long-term BPF supplementation in an animal model resulted in favorable effects on body weight regulation, fasting glucose, and glucose tolerance, reflecting synergistic actions of dietary fiber and phenolic compounds. Increased intake of DF and other bioactive molecules with anti-obesity effects present in BPF that can be achieved by direct diet supplementation, flour fortification, or enrichment of confectionery products, has a perspective in diet modification towards obesity prevention. By combining functional use and nutritional evaluation, this study clearly demonstrated the potential of industrial-scale BPF to improve cereal-based diets, and support obesity prevention strategies. Overall, these findings establish BPF as a scalable, sustainable, and nutrient-dense flour with considerable potential for functional food and nutraceutical applications, supporting future research, commercial development, and the diversification of functional food products. The semi-industrial production of BPF demonstrates the feasibility of scale-up at the case-study factory, while the ongoing cost–benefit (CBA) and life cycle (LCA) analyses will provide essential information on the economic and environmental implications of the process.

## Figures and Tables

**Table 1:** Proximate composition of beetroot pomace flour (BPF), including fiber fractions (soluble, including fructans, and insoluble, including cellulose) and total carbohydrate to total fiber ratio, as well as energy value.

Component	Content (g/100 g)
Fat	0.62 ± 0.07
Protein	12.0 ± 0.3
Available carbohydrates (calculated) *	51.1 ± 1.4
Sugars (determined)	50.6 ± 0.8
Glucose	1.88 ± 1.88
Fructose	0.85 ± 0.85
Sucrose	47.9 ± 0.5
Total dietary fiber	26.7
- Insoluble fiber	16.1 ± 0.5
Cellulose	9.3 ± 0.3
- Soluble fiber	7.1 ± 0.3
Fructans	3.5 ± 0.3
Carbohydrate-to-fiber ratio **	2.9
Energy value ***	1308 kJ (311 kcal)

Values are expressed as mean ± standard deviation (*n* = 3). * Available carbohydrate content was calculated using Equation (1). ** Carbohydrate-to-fiber ratio was obtained by dividing of total content of carbohydrates (available carbohydrate plus total fiber) with total fiber content. *** Energy value in kcal per 100 g of BPF was calculated by multiplying fat with 9 kcal/g, determined carbohydrate and protein with 4 kcal/g, and fiber with 2 kcal/g.

**Table 2:** Glycemic status, body weight gain, water, food and energy intake, food efficiency ratio per food (FER1) and per energy intake (FER2), and glucose tolerance in OGTT (AUC) of C57BL/6J mice upon 150 days of cereal-based diet (CBD) supplemented with 10 mg of beetroot pomace flour (BPF) (CBD-BPF) in comparison to high-fat and sucrose cereal diet without (CBD*) and with 10 mg of BPF addition (CBD* + BPF).

Parameter	CBD	CBD-BPF	CBD*	CBD*-BPF
Fasting glycemia (mmol/L)	8.9 ± 1.6 ^b^	9.2 ± 1.7 ^b^	12.4 ± 1.4 ^a^	8.4 ± 3.7 ^b^
Body weight gain (g)	11.1 ± 2.5 ^a^	6.7 ± 1.9 ^b^	10.8 ± 1.2 ^a^	4.9 ± 3.7 ^b^
Water intake (mL/d)	8.6 ± 0.4 ^a^	8.3 ± 0.2 ^a^	8.4 ± 0.4 ^a^	8.4 ± 0.3 ^a^
Food intake (g/d)	1.92 ± 0.03 ^a^	1.91 ± 0.04 ^a^	1.87 ± 0.05 ^a^	1.91 ± 0.03 ^a^
Energy intake (kcal/d)	5.8 ± 0.1 ^b^	5.7 ± 0.1 ^b^	14.6 ± 0.3 ^a^	14.7 ± 0.1 ^a^
FER1 ** (g/g)	0.038 ± 0.009 ^a^	0.023 ± 0.007 ^b^	0.039 ± 0.005 ^a^	0.017 ± 0.013 ^b^
FER2 *** (×10^−3^ g/kcal)	12.8 ± 2.9 ^a^	7.8 ± 2.2 ^b^	4.9 ± 3.5 ^b^	2.2 ± 1.7 ^c^
AUC (mM*h)	21.6 ± 5.4 ^b^	16.3 ± 3.9 ^b^	29.0 ± 3.9 ^a^	20.6 ± 6.1 ^b^

The values are represented as mean ± SD (glycemia, body weight gain, FER1, FER2 and AUC: *n* = 8; water intake, food intake and energy intake: *n* = 21). Data were subjected to one-way ANOVA (between-subjects factor: food treatment; four levels: CBD, CBD-BPF, CBD* and CBD*-BPF, degree of freedom was three); different superscripts within the same row indicate a significant difference in means, according to Tukey’s HSD test (*p* < 0.05). * Groups exposed to a high-fat and sucrose diet, with or without BPF. ** FER1-food efficiency ratio (weight gain/food intake). *** FER2-food efficiency ratio (weight gain/energy intake).

**Table 3:** Proximate composition of cookies with and without beetroot pomace flour (BPF) along with glycemic index and load, carbohydrate: fiber ratio and % of daily recommendation intake (DRI) per serving size of 35 g.

Parameters	Spelt Cookies with 20% BPF	Spelt Cookies
Available carbohydrate (g/100 g)	50.7 ± 2.0 ^b^	60.0 ± 0.7 ^a^
Total fiber (g/100 g)	7.9 ± 0.6 ^a^	3.4 ± 0.2 ^b^
Carbohydrate fiber ratio *	7.4	18.6
Glycemic index	30 ± 8 ^b^	56 ± 13 ^a^
Glycemic load	5.3	11.8
DRIs for total fiber **	10	4

* Total carbohydrate/total fiber (Total carbohydrate: available carbohydrate plus total fiber). ** Recommendation for fiber intake 28 g/day. Different lowercase letters within the same row indicate a significant difference in means, according to the t-test (*p* < 0.05).

**Table 4:** Texture properties of spelt cookies with (enriched cookies) and without addition of 20% of beetroot pomace flour (BPF) (control).

Sample	Hardness (g)	Elasticity (mm)	Work of Compression (mJ)
Control cookies	7138.5 ± 1038.8 ^a^	0.917 ± 0.023 ^b^	1771.0 ± 250.2 ^b^
Enriched cookies	6002.2 ± 1098.5 ^a^	1.682 ± 0.190 ^a^	4754.0 ± 924.8 ^a^

Values are expressed as mean ± standard deviation (*n* = 3). Different superscript letters within the same column indicate statistically significant differences (*p* < 0.05) according to the t-test.

## Data Availability

The original contributions presented in this study are included in the article/Supplementary Material. Further inquiries can be directed to the corresponding authors.

## Supplementary Materials

The following supporting information can be downloaded at: https://www.mdpi.com/article/10.3390/foods15071142/s1, Table S1: Sensory evaluation form.
