# Supplementary material for: Valorization of Beetroot Pomace as a Flour Fortifier, Functional Ingredient and Dietary Supplement
Source: Foods. 2026 Mar 26;15(7):1142. doi: 10.3390/foods15071142 (PMC13072988; doi:10.3390/foods15071142)
Supplement: Supplementary file 1 [file foods-15-01142-s001.zip › foods-4021137-supplementary.pdf]

Table S1 SENSORY EVALUATION FORM

Sample code: __________

By marking the appropriate box with an **X**, please indicate the extent to which you like the cookie made with beetroot pomace flour. When evaluating the product, please take into account that it is a **natural product**, free from artificial colors and flavors, and **high in dietary fiber**. If you have any comments or suggestions, you may note them after each question or in the margins.

1. To what extent do you like the taste of the cookie with added beetroot pomace flour?

Not at all | do not like it | do not like it | neither like nor dislike | like it | like it very much

☐ ☐ ☐ ☐ ☐ ☐ ☐ ☐ ☐

2. To what extent do you like the texture of the cookie with added beetroot pomace flour?

Not at all | do not like it | do not like it | neither like nor dislike | like it | like it very much

☐ ☐ ☐ ☐ ☐ ☐ ☐ ☐ ☐

If you do not like the texture, please circle the property that bothers you the most: hardness, crispiness, crumbliness, chewiness, or describe in your own words what bothers you:

3. To what extent do you like the color of the cookie with added beetroot pomace flour?

Not at all | do not like it | do not like it | neither like nor dislike | like it | like it very much

☐ ☐ ☐ ☐ ☐ ☐ ☐ ☐ ☐

4. To what extent do you like the aroma of the cookie with added beetroot pomace flour?

Not at all| do not like it | do not like it | neither like nor dislike | like it | like it very much

☐ ☐ ☐ ☐ ☐ ☐ ☐ ☐ ☐

5. To what extent does the cookie satisfy your need for sweetness?

Does not satisfy at all | insufficiently | neither satisfies nor dissatisfies | satisfies | completely satisfies

☐ ☐ ☐ ☐ ☐ ☐ ☐ ☐ ☐

6. To what extent do you like the cookie overall?

Not at all | do not like it | do not like it | neither like nor dislike | like it | like it very much

☐ ☐ ☐ ☐ ☐ ☐ ☐ ☐ ☐

Please mark your gender and age group with an X, and then answer the remaining questions in the same way.

Gender: Male _______ Female _______

Age: 15–25 years _____ 25–35 years _____ 35–50 years _____ >50 years _____

Do you follow a special diet? __________________________

If yes, please state the reason for following a special diet: __________________________

2.1 Do you eat sweets?

Often ☐ Occasionally ☐ Rarely ☐

2.2 Do you eat cookies?

☐ Yes ☐ No

2.3 Do you prefer wholegrain products without additives?

☐ Yes ☐ No ☐ No preference

2.4 When purchasing food, do you pay attention to the dietary fiber content?

☐ Yes ☐ No ☐ No preference

2.5 Would you buy the cookie you have just tasted?

No | Unlikely | Not sure | Likely | Yes

2.6 What would be decisive in your decision to buy the cookie you have just tasted?

☐ Positive health effects

☐ High dietary fiber content from beetroot

☐ Absence of artificial colors and flavors

☐ Taste

2.7 Does knowing that the cookie contains a by-product remaining after juice extraction influence your decision to buy it?

☐ Yes ☐ No

All suggestions related to the product name or ways to improve the product are welcome:
